# Paroxysmal Cold Hemoglobinuria Associated With Monoclonal B-Cell Lymphocytosis in an Elderly Patient: A Report of a Rare Case

**DOI:** 10.7759/cureus.98102

**Published:** 2025-11-29

**Authors:** Summiya Nasim, Matthew Bartock

**Affiliations:** 1 Internal Medicine, Parkview Health, Fort Wayne, USA; 2 Parkview Packnett Family Cancer Institute, Parkview Health, Fort Wayne, USA

**Keywords:** autoimmune hemolytic anemia, cold agglutinin disease, complement mediated hemolysis, donath-landsteiner antibody, monoclonal b-cell lymphocytosis, paroxysmal cold hemoglobinuria

## Abstract

We report the case of an 86-year-old male with chronic anemia who presented with indirect hyperbilirubinemia, undetectable haptoglobin, and reticulocytosis. The peripheral smear showed no schistocytes. The cold-agglutinin titer was mildly positive, and the Donath-Landsteiner antibody test was positive. Bone marrow biopsy revealed a small CD5⁺ B-cell clone consistent with monoclonal B-cell lymphocytosis (MBL). The patient was diagnosed with paroxysmal cold hemoglobinuria (PCH) associated with MBL and treated with rituximab, achieving clinical stability without transfusion requirement. This case illustrates a rare coexistence of PCH and MBL in an elderly patient and emphasizes the importance of comprehensive hematologic evaluation for unexplained chronic hemolysis.

## Introduction

Paroxysmal cold hemoglobinuria (PCH) is an underrecognized form of autoimmune hemolytic anemia (AIHA). It is characterized by complement-mediated intravascular hemolysis due to the presence of the Donath-Landsteiner (DL) antibody. This biphasic IgG autoantibody binds to red blood cells (RBCs) at low temperatures and triggers complement fixation and hemolysis upon rewarming to 37°C. While PCH was historically more common in children following viral infections, its occurrence in adults remains exceptionally uncommon and diagnostically challenging. It accounts for less than 5% of AIHA cases and is frequently underrecognized in elderly populations, often being misclassified as cold agglutinin disease (CAD) because of overlapping features but distinct pathophysiologic mechanisms [1,2].

Monoclonal B-cell lymphocytosis (MBL) is an asymptomatic premalignant clonal B-cell disorder that may precede chronic lymphocytic leukemia (CLL) and is increasingly detected in elderly individuals during hematologic evaluation [3,4]. Although typically indolent, emerging evidence suggests that MBL may contribute to immune dysregulation and the development of autoimmune cytopenias. The coexistence of PCH with B-cell clonal disorders, including MBL, is exceedingly rare, and the underlying immunologic relationship between clonal B-cell activity and autoantibody-mediated hemolysis remains poorly understood [5].

Here, we describe a unique case of PCH associated with MBL in an elderly male. This report highlights an unusual clinical overlap that underscores the role of comprehensive hematologic assessment, including antibody testing and bone marrow evaluation, in patients with unexplained or chronic hemolysis.

## Case presentation

An 86-year-old male with a past medical history of paroxysmal atrial fibrillation, hypertension, transient ischemic attack, benign prostatic hyperplasia, and chronic anemia presented to the hematology clinic for further evaluation of longstanding anemia. He reported only chronic fatigue without associated symptoms such as jaundice, hematuria, weight loss, fevers, or bleeding. There were no recent infections, medication changes, or systemic complaints.

Initial laboratory evaluation revealed hemoglobin levels ranging from 8.9 to 12.0 g/dL over several years, with persistently undetectable haptoglobin, elevated indirect bilirubin, and reticulocytosis, consistent with chronic hemolysis. A summary of key laboratory parameters is presented in Table 1.

**Table 1:** Summary of Laboratory Findings LDH, lactate dehydrogenase; TIBC, total iron-binding capacity; PCH, paroxysmal cold hemoglobinuria.

Parameter	Patient Result	Reference Range
Hemoglobin	8.9–12.0 g/dL	13.5–17.5 g/dL
Reticulocyte count	Elevated	0.5–2.5 %
Total bilirubin	Elevated (indirect)	0.2–1.2 mg/dL
Haptoglobin	Undetectable	30–200 mg/dL
LDH	Elevated	140–280 U/L
Cold agglutinin titer	1:64	< 1:32
Donath–Landsteiner antibody	Positive	Negative
Peripheral smear	Normocytic anemia, no schistocytes	—
Iron studies	Normal	Serum iron 50–150 µg/dL, TIBC 250–450 µg/dL
Vitamin B12/folate	Normal	B12 200–900 pg/mL, folate 2–20 ng/mL

Iron, vitamin B12, and folate levels were within normal limits. Peripheral smear showed normocytic anemia without schistocytes or burr cells. Direct antiglobulin test (DAT) was initially negative, but a super DAT demonstrated cold agglutinins (titer 1:64). Donath-Landsteiner antibody testing was positive. Bone marrow biopsy revealed a small clonal CD5+, kappa-restricted B-cell population consistent with MBL. The patient was diagnosed with PCH associated with MBL. He was treated with weekly rituximab infusions for four weeks. No intravenous immunoglobulin (IVIG), steroids, or transfusions were required. He remained clinically stable throughout treatment and follow-up.

## Discussion

This case illustrates the coexistence of two rare entities: PCH and MBL. PCH in adults is seldom reported and is classically post-infectious in children. Diagnosis relies on demonstration of the Donath-Landsteiner antibody, even when the standard DAT is negative. The DL antibody is an IgG autoantibody directed against the P antigen on RBCs; it binds at cold temperatures and fixes complement, leading to hemolysis as cells are rewarmed to 37 °C. This biphasic mechanism distinguishes PCH from cold agglutinin disease, which is IgM-mediated.

MBL is typically asymptomatic but can influence immune tolerance and autoantibody production. In our patient, this clonal B-cell process may have served as an immunologic trigger for the DL antibody response [3]. Similar immune dysregulation involving clonal B-cell proliferation has been described in autoimmune hemolytic anemias, supporting B-cell involvement in pathogenesis [4]. Rituximab was selected because of its efficacy in antibody-mediated disorders [4]. However, the mainstay of PCH management remains in cold avoidance and treatment of the underlying infection, with immunotherapy reserved for chronic or relapsing disease. The favorable response to rituximab alone supports B-cell involvement in pathogenesis [5].

This report has been prepared in accordance with the CARE guidelines to ensure transparency and educational value [2].

## Conclusions

PCH associated with MBL is an exceptionally rare entity that broadens the spectrum of cold mediated hemolytic disorders in adults. Clinicians should consider PCH in elderly patients with chronic hemolysis and negative standard DAT results. Recognition of this association permits timely diagnosis and directed therapy, with excellent outcomes when identified early.
